# Effect of Different Laser Groove Texture Collation Frequency on Tribological Properties of 0Cr17Ni7Al Stainless Steel

**DOI:** 10.3390/ma15134419

**Published:** 2022-06-22

**Authors:** Liguang Yang, Wensuo Ma, Fei Gao, Shiping Xi

**Affiliations:** 1 School of Mechatronics Engineering, Henan University of Science and Technology, Luoyang 471023, China; bmzgedu@126.com; 2 Luoyang Bearing Research Institute Co., Ltd., Luoyang 471039, China; yejinxsp@163.com; 3 School of Mechanical Engineering, Tsinghua University, Beijing 100084, China; 13698807332@126.com

**Keywords:** nanosecond, picosecond, texture groove, collision frequency, tribology

## Abstract

Laser surface texture is very effective in antifriction systems, but its applications and research in dry friction are not enough. In this study, the groove texture was prepared on the surface of 0Cr17Ni7Al stainless steel, a common material of sliding bearing, by nanosecond and femtosecond laser, respectively. The tribological properties of the two kinds of laser groove textures with different collision frequencies were studied in depth. The results show that the friction coefficients of groove texture prepared by nanosecond and picosecond lasers are lower than that of the untextured surface. The antifriction characteristics of the laser texture are very good. The average friction coefficient of nanosecond texture at the rotation radius of 15 mm is Z = 0.7318. The best friction-reducing effect is achieved. In general, the friction coefficient of nanosecond texture is lower than that of picosecond texture. When the friction radius is 22.5 mm and the number of collisions is 24,000, the lowest picosecond texture wear rate is *H* = 3.342 × 10^−4^ mm^3^/N·mm. However, when the radius is 15 mm and the collision frequency is 36,000 times, the wear rate of nanosecond texture reaches the highest *H* = 13.680 × 10^−4^ mm^3^/N·mm. The wear rate of the untextured surface has been exceeded. It can be seen that not all rotation radius textures are more wear-resistant than untextured surfaces. In addition, nanosecond groove texture and picosecond groove texture seem to produce different tribological properties. It is found that, under the same friction experimental conditions, different collision frequencies will affect the friction and wear properties of nanosecond and picosecond groove-textured surfaces.

## 1. Introduction

In the 1990s, Etsion [1] took the lead in studying the laser texture technology and applied the laser surface texture technology to mechanical seals. His research results have become an important reference in related fields. To date, scholars’ research on bionics has gradually become a hot topic. The friction characteristics of the surface can be effectively improved by machining the texture with regular geometric dimensions and an orderly arrangement on the part surface. Surface modification has been widely used in many fields; for example, in the field of material surface friction reduction [2,3,4] and friction pair surface wear resistance [5,6], on some special occasions where surface friction needs to be increased [7], in environments where the system needs to reduce vibration [8,9,10], in the special field of resistance to adhesion [11], in the special environment of anti-creep [12], with long-term low-temperature equipment [13], and when the equipment needs to increase the bearing capacity [14], in the field of maintaining wettability [15], as well as areas with functional hydrophobicity requirements [16] and scenarios with increased adhesion [17]. It has shown good application prospects and become an effective way to achieve the high efficiency, miniaturization and high reliability of mechanical equipment [18].

With the development of surface modification technology, some scholars have studied surface-texture processing technology. Surface texture is a technology that uses laser processing, electron beam etching and other methods to process the specific micro-morphology on the part’s surface. This surface-treatment process involves material properties and interface effects [19,20,21,22,23,24]. Generally speaking, the micro-texture morphology imitates and optimizes the bionic structure to achieve the aims of industrial production. However, to reduce the processing cost and improve the feasibility, the complex bionic structure is often simplified into pits and grooves in the research. Among them, the laser preparation of surface texture has the advantages of a fast processing speed and high efficiency. Moreover, the non-contact machining method makes it easier to realize precision machining and causes less damage to the surface. In recent years, this has attracted extensive attention. Common texture morphologies include pit shape, groove shape, convex shape and scale shape [25,26]. Domestic and foreign scholars have studied the effects of different texture morphologies on friction characteristics and lubrication effect. The friction and wear conditions are improved, and the service life of mechanical parts is effectively prolonged. At present, a laser surface micro-texture is widely used in cutting tools, engine cylinders, bearings, medicine and other fields [27].

The factors affecting the tribological characteristics of the friction pair surface include relative speed, load, lubricating medium, surface characteristics, temperature field and metal matrix composites [28,29,30,31]. Surface property is one of the key factors. However, at this stage, the research on surface properties is not sufficient. In this paper, two laser methods with high processing efficiency were used to prepare the groove texture on the surface of the 0Cr17Ni7Al material, which are commonly used in sliding bearings [32]. Under different collision frequencies between ball and groove, the friction and wear experiments of groove texture were carried out by MFT-5000 friction and wear tester. The surface morphology, friction and wear state of the samples were analyzed by a three-dimensional white light interferometer, scanning electron microscope (SEM) and energy-dispersive spectrometer (EDS). The characteristics of the different collision frequencies between ball and disk under different rotation radii were revealed. The wear mechanism of the friction pair with groove texture was discussed. The tribological behavior of the groove-textured surface was studied under different groove contact frequencies. The effect of collision frequency on the friction reduction and wear resistance of different laser groove-textured surfaces was mastered. It is of practical significance to select the optimal groove texture to guide the engineering applications of antifriction and wear resistance.

## 2. Materials and Methods

### 2.1. Processing Laser Plate

Laser processing is used to irradiate the laser beam on the workpiece surface. The laser beam interacts with the material to make the workpiece produce high-temperature melting, and is thrown out by the shock wave under the photothermal effect with the high energy of the laser to realize the cutting, surface treatment, drilling and micro-machining of materials (including metals and non-metals).

**Figure 1:**
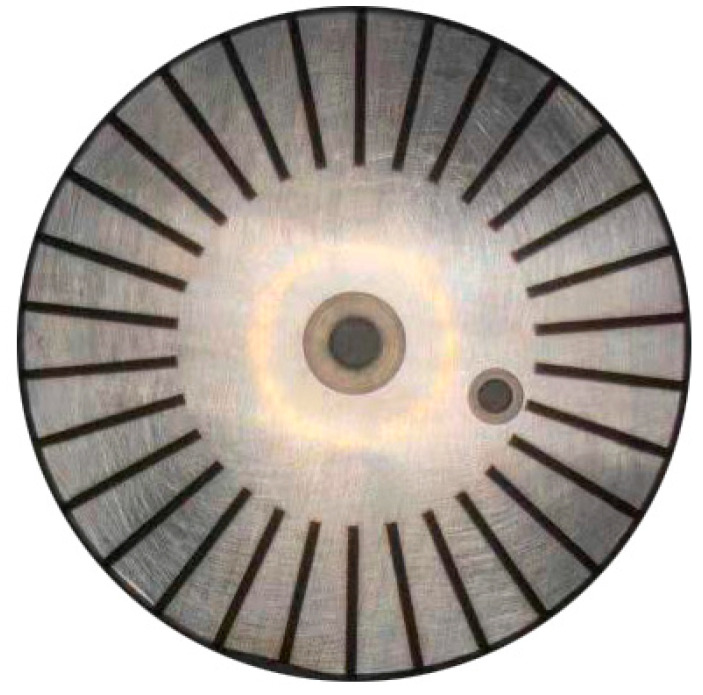
Nanosecond texture.

**Figure 2:**
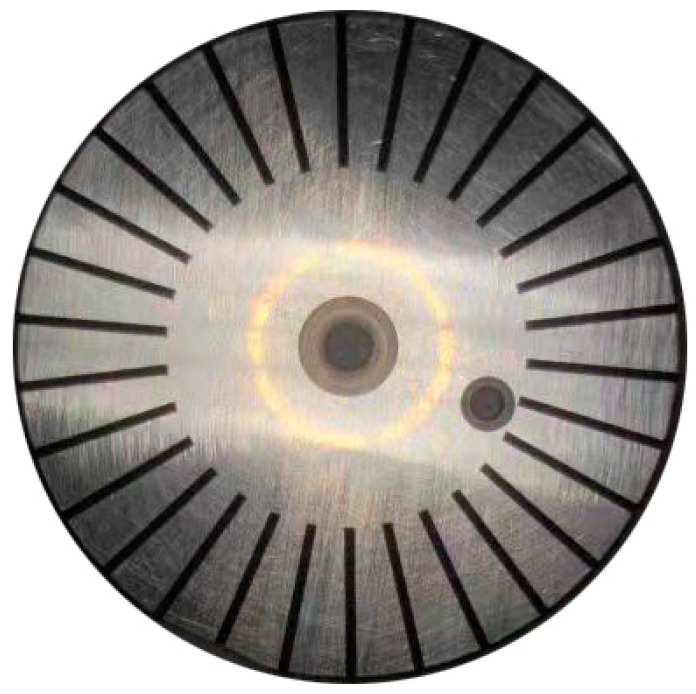
Picosecond texture.

Table 1 and Table 2 show the specific parameters of nanosecond laser and picosecond laser equipment, respectively. Surface grooves with a width of 800 µm, depth of 150 µm, length of 12 mm and 30 grooves were processed, as shown in Figure 1 and Figure 2, respectively. Nanosecond-laser surface-texture and picosecond-laser surface-texture samples were provided by Shenzhen transcend laser Intelligent Equipment Co., Ltd. (Shenzhen, China).

### 2.2. Friction and Wear Test Material

**Figure 3:**
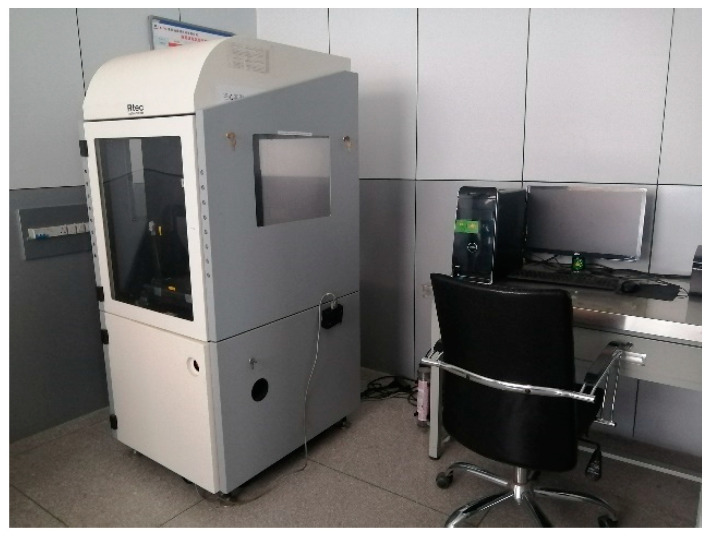
MFT-5000 friction and wear test bench.

The friction characteristics of laser groove texture on 0Cr17Ni7Al surface were experimentally studied using friction and wear testbed (MFT-5000). The testing machine is shown in Figure 3, and the relevant parameters of the test piece shown in Table 3. The relative position of the friction pair in the friction and wear test is shown in Figure 3. The experimental parameters are shown in Table 4.

Two different test pieces were prepared and compared during the experiment: nanosecond-laser surface-texture and picosecond-laser surface-texture. Before the test of each sample, acetone was used for further ultrasonic cleaning for 5 min to wipe the surface of the test piece. After the test, the wear morphology was detected by white-light interferometer (manufactured by Rtec instruments), scanning electron microscope (manufactured by Carl Zeiss SMT Co., Ltd.) and energy spectrum (manufactured by Oxford Instruments Nanotechnology). The experimental situation was analyzed, combined with the change in friction coefficients.

### 2.3. Friction and Wear Calculation

The specific experimental parameters are listed in Table 4. The white-light interferometer was used to detect the cross-sectional profile of the wear mark; the wear area was calculated by integrating the cross-sectional profile; the wear volume was obtained by multiplying the friction step and wear area [33]:

(1) $$H=\frac{V}{F\cdot S}$$

where *H* is the wear rate, 10^−4^ mm^3^/N·mm; *V* is the wear volume, mm^3^; *F* is the normal load, N; *S* is the running distance, mm. In this study, the error is reduced by the average value of the wear rate of three parallel tests.

## 3. Results and Discussion

### 3.1. Friction Coefficient and Wear Rate

**Figure 4:**
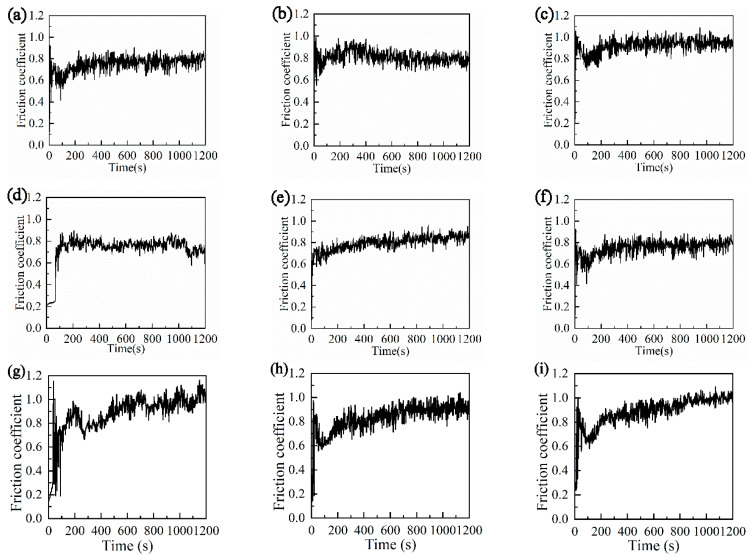
Friction coefficient curves of different samples: (**a**) nanosecond texture rotation radius 15 mm; (**b**) nanosecond texture rotation radius 18 mm; (**c**) nanosecond texture rotation radius 22.5 mm; (**d**) picosecond texture rotation radius 15 mm; (**e**) picosecond texture rotation radius 18 mm; (**f**) picosecond texture rotation radius 22.5 mm; (**g**) untexture rotation radius 15 mm; (**h**) untextured rotation radius 18 mm; (**i**) untextured rotation radius 22.5 mm.

As the previous study [33] shows, with the progress of friction, the untextured surface is difficult or takes a longer time to enter stable wear. Regardless of the friction radius of the untextured surface, the friction increases with the friction time. As can be seen from Figure 4, under the same friction conditions, most textured surfaces quickly enter the stable wear stage at 200 s. After reaching the stable wear stage, the groove texture can be stabilized within a small variation range. It does not change greatly with the increase in friction time. Figure 4 and Table 5 show that the friction coefficients of nanosecond texture and picosecond texture are smaller than those of the untextured surface. It can be seen that the groove texture has a good antifriction performance.

The reason that the friction coefficient of the groove texture is lower than that of the untextured surface may be due to the following three aspects. Firstly, this may be caused by the wear debris on the surface of the groove texture being taken away with friction and captured and stored by the groove texture during the friction process, which reduces the further aggravation of wear particles. Secondly, the ball collides with the groove continuously to result in friction-pair vibration, which causes wear debris roll and reduces the friction. Thirdly, the existence of texture grooves can reduce the contact area and contact time of the friction pair in the friction process, reduce the increase in friction heat, achieve heat dissipation, and slow down the formation of oxidative wear and fretting wear. Under the same test conditions, the friction coefficient of the untextured surface gradually increases with the progress of the friction experiment. Under the same preparation method, the variation in texture friction coefficient with a different friction radius may be related to the collision frequency of the texture in the friction process.

Combined with Table 5 and Table 6, the friction coefficients of nanosecond texture and picosecond texture increase with the increase in friction radius and the decrease in collision frequency between ball and groove. Table 6 shows the collision frequency of friction pair in the friction process under different rotation radius, with the same siding linear speed, time and test load. Under a nanosecond texture in the overall friction process with a rotation radius of 15 mm, the ball collided with the groove 36,000 times. At this time, the minimum average friction coefficient of *Z* = 0.7318 was reached. This shows that the best antifriction effect was achieved at this time.

As can be seen from Table 5 and Table 6, the influence of collision frequency on the texture of the grooves prepared by different lasers are the same. The friction coefficients of nanosecond texture and picosecond texture decreased with the decrease in collision frequency. In general, the friction coefficient of nanosecond texture is lower than that of the picosecond texture. The average friction coefficient was *Z* = 0.8425 at the rotation radius of 22.5 mm.

Combined with Table 5 and Table 6, with the increase in friction radius, the collision frequency decreases, and the nanosecond texture and picosecond texture show a downward trend. The wear rate of picosecond texture under each rotation radius was lower than that of nanosecond texture. When the friction radius was 22.5 mm and the collision times were 24,000, the lowest picosecond texture wear rate was *H* = 3.342 × 10^−4^ mm^3^/N·mm. However, when the radius was 15 mm and the collision frequency was 36,000 times, the wear rate of nanosecond texture reached its highest, *H* = 13.680 × 10^−4^ mm^3^/N·mm. The wear rate of the untextured surface in the previous study [31] was exceeded. Not all textures with rotation radius are more wear-resistant than untextured surfaces. Therefore, we should find the friction and wear law of texture. Only by making rational use of the collision frequency can the surface texture modification be applied to engineering practices. This can not only reduce friction, but also play a role in wear resistance; otherwise, it will lead to a poor use effect.

### 3.2. Worn-Morphology Analysis

After the friction experiment, the flaked convex part on the surface of the friction pair is called the hard-phase peak [33]. The hard-phase peak may be caused by the migration of wear debris under the combined action of load and sliding speed during the friction process. Heat is rapidly generated during friction, and is rapidly cooled during the friction pair collision. Under this repeated action, it is formed by gluing and “cold welding”. According to the position of the hard-phase peak, it can be divided into a wear-mark hard-phase peak and wear-mark-edge hard-phase peak. The hard-phase peak at the edge of the wear mark helps to form a supporting effect on the surface of the friction pair, which could protect the surface from further wear. The hard-phase peak of the wear mark will further aggravate the wear groove effect and may even play an important role in the evolution from abrasive wear to adhesive wear.

**Figure 5:**
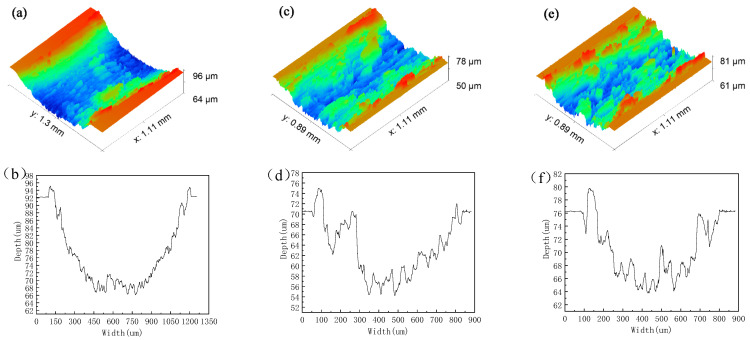

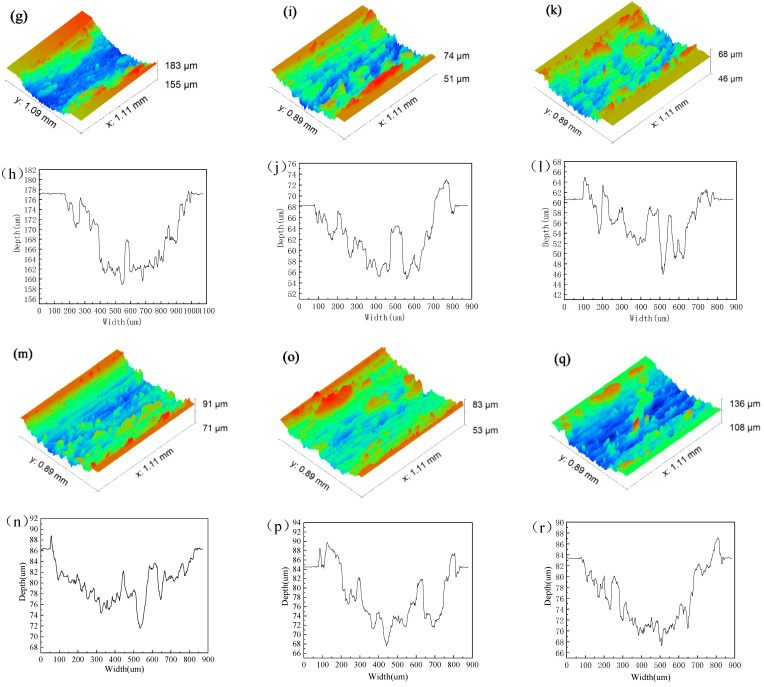
Morphology and depth of wear marks of different samples: (**a**,**b**) nanosecond texture rotation radius 15 mm; (**c**,**d**) nanosecond texture rotation radius 18 mm; (**e**,**f**) nanosecond texture rotation radius 22.5 mm; (**g**,**h**) picosecond texture rotation radius 15 mm; (**i**,**j**) picosecond texture rotation radius 18 mm; (**k**,**l**) picosecond texture rotation radius 22.5 mm. (**m**,**n**) untextured rotation radius 15 mm; (**o**,**p**) untextured rotation radius 18 mm; (**q**,**r**) untextured rotation radius 22.5 mm.

The three-dimensional wear trace morphology and wear trace depth curve in Figure 5 show that, under different friction radius, the texture surface is dominated by the hard-phase peak at the edge of the wear trace, and supplemented by the hard-phase peak of the wear trace. This is mainly due to the wear debris in the middle of the wear mark on the textured surface during the friction process, especially the large volume of wear debris that falls into the groove. The wear debris at the wear mark is greatly reduced. The wear debris at the edge of the wear mark cannot be captured by the texture, and the edge hard-phase peak is formed at the edge of the wear mark. The small volume of wear debris at the wear mark forms the hard-phase peak of the wear mark.

The wear mark depth curve show that if there are more hard-phase peaks on the edge of the wear mark and fewer hard-phase peaks on the wear mark, the optimal antifriction effect will be achieved. That is to say, when there is both a wear mark hard-phase peak and an edge hard-phase peak, the reverse effect of the wear mark hard-phase peak is much greater than the positive effect of the edge hard-phase peak. There is a hard phase peak in the wear scar on the untextured surface.

### 3.3. Scanning Electron Microscope Analysis of the Worn Surface

**Figure 6:**
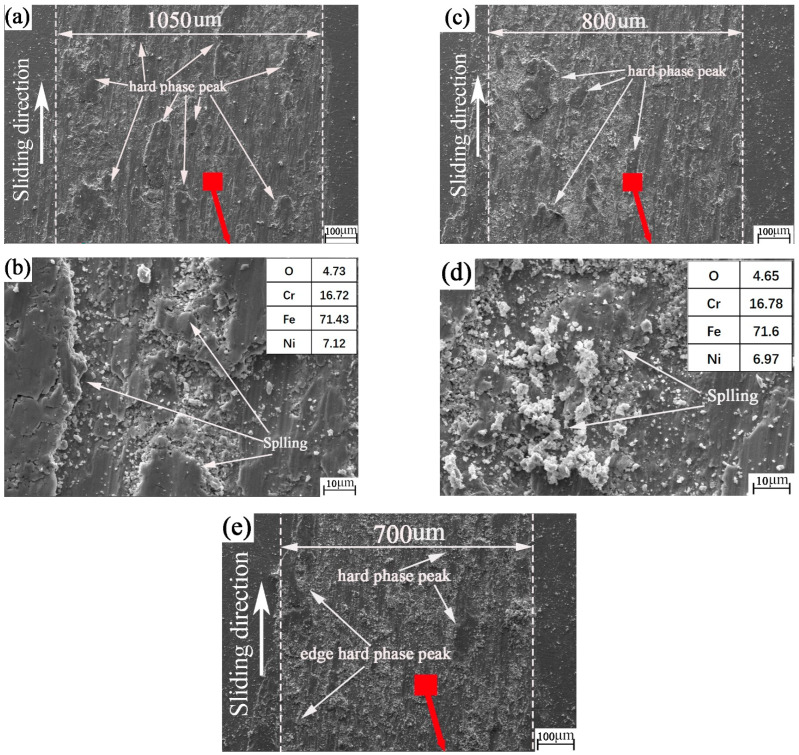

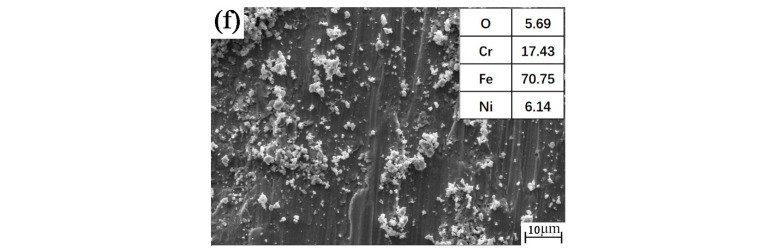
SEM and EDS images of the nanosecond textured worn plates: (**a**,**b**) nanosecond texture rotation radius 15 mm; (**c**,**d**) nanosecond texture rotation radius 18 mm; (**e**,**f**) nanosecond texture rotation radius 22.5 mm.

As shown in Figure 6a–f, there is both wear debris and flake cement on the nanosecond texture surface. This shows that the nanosecond textured surface is undergoing the common development stage of abrasive wear and adhesive wear. Figure 6a–d show that the size and number of wear hard-phase peaks on the nanosecond texture surface are larger than those on the edge. The two hard-phase peaks are separated from each other and are far away. They have no impact on each other. At this time, the hard-phase peak of the wear mark plays a decisive role and is at the stage of intensified wear. As shown in Figure 6e,f, at this time, this is mainly abrasive wear. There are edge hard-phase peaks on both sides of the wear mark. The number of hard-phase peaks in wear marks is small and the volume is small. Therefore, the antifriction effect is the main effect.

The element content on the surface of the plate sample in Figure 6 and the chemical composition of the plate sample in Table 7 are combined. Oxygen was found on the surface of the plate sample. It can be inferred that oxidation corrosion wear occurred on the nanosecond textured surface during the friction process. However, it is difficult to judge whether there is material transfer between the plate and the ball in the process of friction. Further analysis of the energy spectrum later in this paper is required.

**Figure 7:**
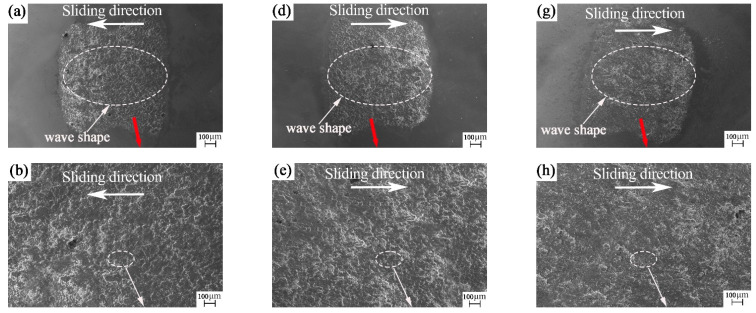

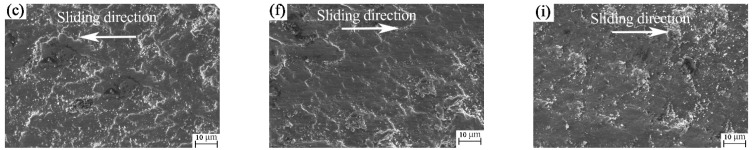
SEM images of the nanosecond textured worn balls: (**a**–**c**) rotation radius 15 mm; (**d**–**f**) rotation radius 18 mm; and (**g**–**i**) rotation radius 22.5 mm.

According to the SEM of the ball surface from Figure 7a–i, the wear mark of the ball is quadrilateral. The main reason for this is that, during the wear process, the ball collides with the groove and the edge of the groove is equivalent to a sharp edge, which cuts the wear mark of the ball into a straight line and then becomes a quadrilateral. Figure 7a,d,g show that the wear marks appear as individual waves along the sliding direction. This may be because the ball will encounter 30 collisions with the groove texture for each revolution during the friction process. Under the combined action of pressure, speed in the moving direction and friction heat, the wear debris is impact-glued on the wear-mark surface of the ball. In the friction process, these layered waves will achieve the same effect as windsurfing on the sea, which is conducive to reducing the resistance on the surface of the friction pair. It can be seen from Figure 7c,f,i that there is a small amount of fine grinding debris at the wear mark on the ball surface. This may be because, at the end of friction, the wear debris on the disk surface adhered to the ball surface due to the action of pressure. This further confirms our judgment on the formation of wave-layered wear marks caused by the transfer of wear debris.

**Figure 8:**
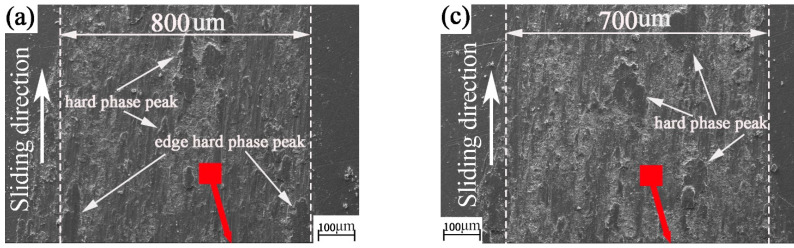

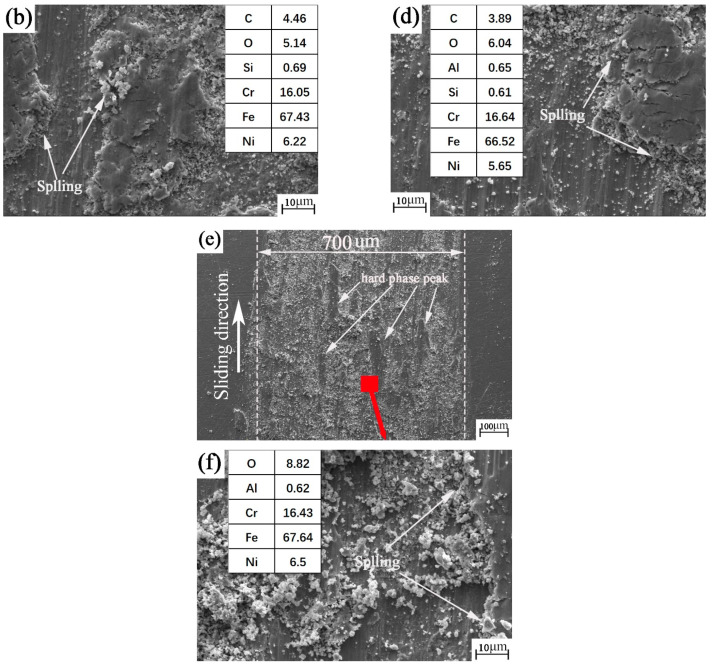
SEM and EDS images of the picosecond textured worn plates: (**a**,**b**) picosecond texture rotation radius 15 mm; (**c**,**d**) picosecond texture rotation radius 18 mm; (**e**,**f**) picosecond texture rotation radius 22.5 mm.

As can be seen from Figure 8a–f, there is both wear debris and flake cement on the surface of picosecond texture. This shows that the picosecond textured surface is in the common development stage of abrasive wear and adhesive wear. It can be seen from Figure 8a,b that flake glues (i.e., hard-phase peaks) are distributed from the edge of the wear mark to the wear mark area. As can be seen from Figure 8a–d, the size and number of hard-phase peaks of wear marks on the surface of picosecond texture are larger than those on the edge. At this time, the hard-phase peak of wear mark plays a decisive role, and the wear intensifies. As shown in Figure 6e,f, this is mainly abrasive wear. The number of hard-phase peaks in wear marks is small and the volume is small. Therefore, the antifriction effect is the main effect.

Comparing Figure 8 with Figure 6, the oxygen content on the surface of picosecond plate sample increases with the increase in rotation radius. This may be because when the rotation radius is large, the wear mark surface of the disc contacts the air for longer. Therefore, oxygen in the air can become more involved in oxidative corrosion and wear. The oxidation corrosion wear becomes more serious with the increase in rotation radius. The law of oxidation corrosion wear with different nanosecond textures and picosecond textures may be related to the influence of the two different laser preparation methods on the performance parameters of the surface texture. However, it is still difficult to judge whether the material transfer from the plate to the ball occurs during the friction process.

**Figure 9:**
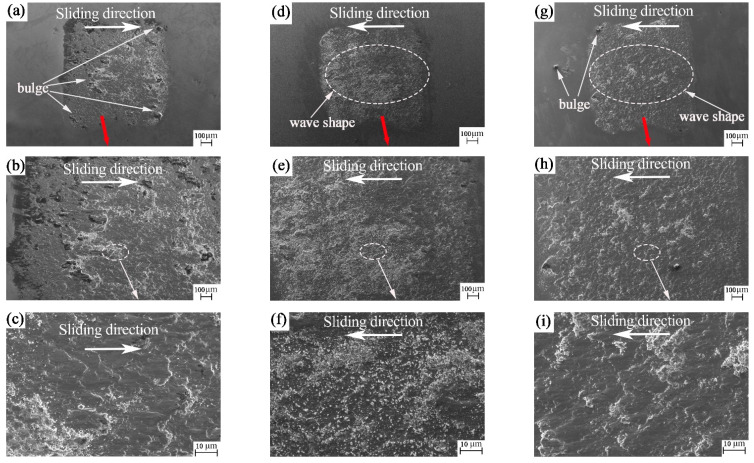
SEM images of the picosecond textured worn balls: (**a**–**c**) rotation radius 15 mm; (**d**–**f**) rotation radius 18 mm and (**g**–**i**) rotation radius 22.5 mm.

From the SEM of the ball surface in Figure 9a–i, the wear mark of the ball is still quadrilateral. Figure 9a,d,g show that the wear marks are similar to sea waves, forming sea waves individually along the sliding direction. This may be because, during the friction process, the ball will encounter 30 collisions with the groove texture for each revolution. Under the combined action of pressure, speed in the moving direction and friction heat, the wear debris is impact-glued on the wear mark surface of the ball. These layered waves will achieve the same effect as windsurfing on the sea during the friction process, which is conducive to reducing the resistance on the surface of the friction pair. Some bulges can be found in Figure 9a. These protrusions cut the plate sample like a sharp sword during the friction process. Figure 9c,f,i show a small amount of fine grinding debris at the wear mark on the ball surface. This may be because, at the end of friction, the wear debris on the plate surface adhered to the ball surface due to the pressure. This further confirms our judgment regarding the formation of wave-layered wear marks caused by the transfer of wear debris.

**Figure 10:**
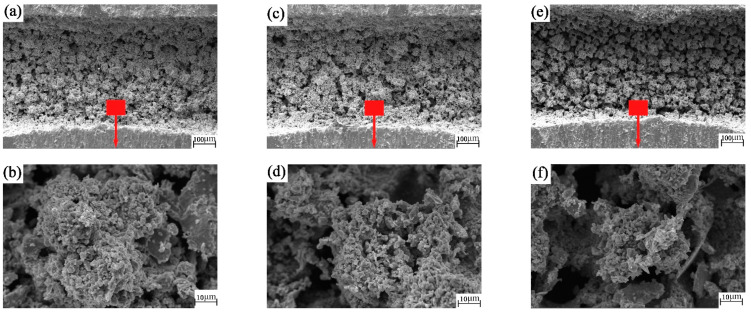

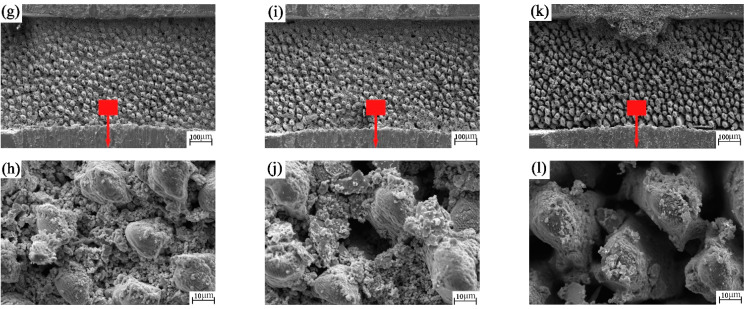
SEM images of the groove textured worn plates: (**a**,**b**) nanosecond texture rotation radius 15 mm; (**c**,**d**) nanosecond texture rotation radius 18 mm; (**e**,**f**) nanosecond texture rotation radius 22.5 mm; (**g**,**h**) picosecond texture rotation radius 15 mm; (**i**,**j**) picosecond texture rotation radius 18 mm; (**k**,**l**) picosecond texture rotation radius 22.5 mm.

From Figure 10a–f, the bottom of the groove with nanosecond texture presents the remelting layer left after processing, in the form of granular slag. The remelted layer at the bottom of the picosecond texture groove in Figure 10g–l shows fine particles. In terms of the processed groove quality, the picosecond texture is finer. The grooves all play roles in the capture and storage of wear debris. As shown in Figure 10a,c,e,g,i,k, there are obvious marks of collision and cutting between the ball and the groove at the edge of the groove, which further confirms our judgment that the quadrilateral wear mark of the groove-texture ball is due to the ball being cut by the edge of the groove.

### 3.4. Energy-Spectrum Analysis of Worn Surface

It can be seen from Table 7 that the chemical composition content of the upper and lower pairs of grinding parts are very similar. In terms of Ni and Al content, the content of 0Cr17Ni7Al is higher than that of 9Cr18, so it can be used as a key element.

**Figure 11:**
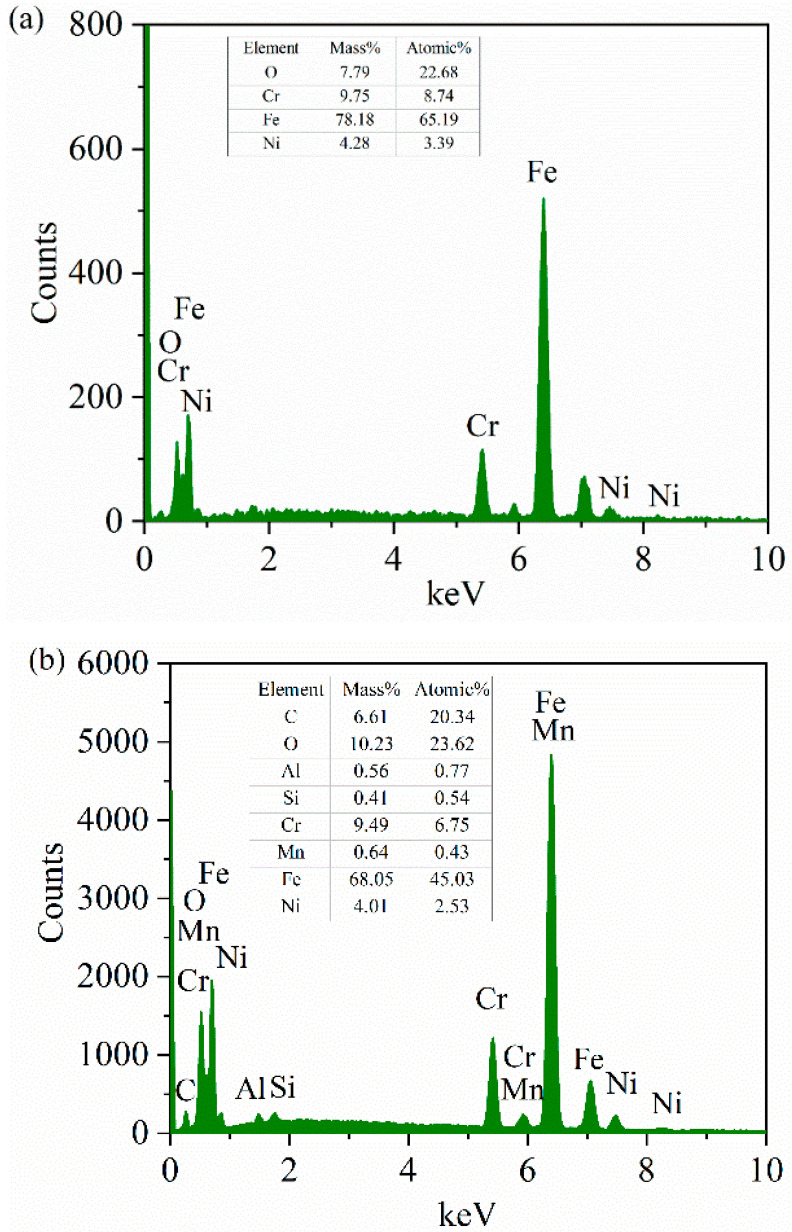

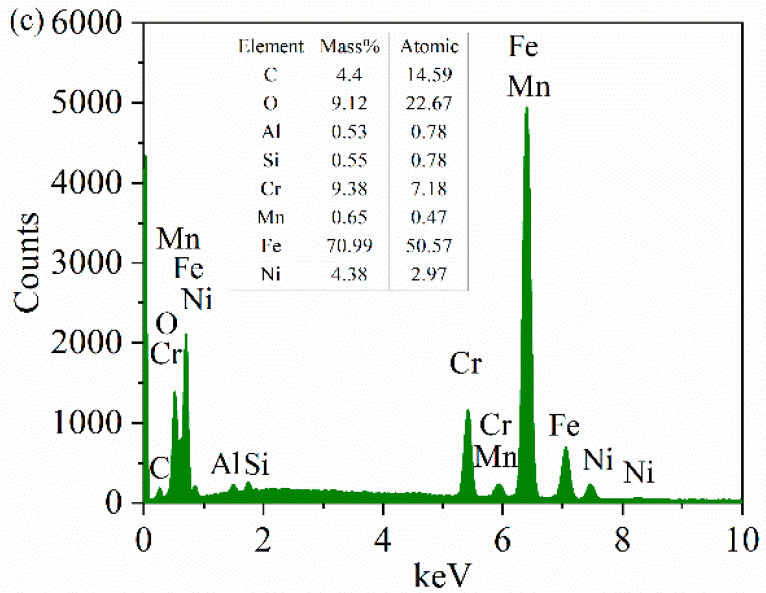
Comparison of EDS spectra of the nanosecond textured worn balls: (**a**) rotation radius 15 mm; (**b**) rotation radius 18 mm; and (**c**) rotation radius 22.5 mm.

**Figure 12:**
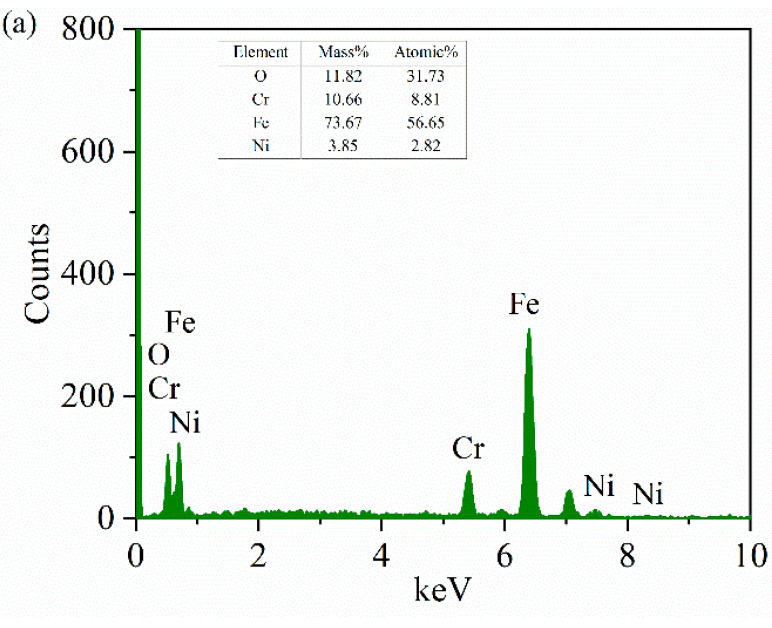

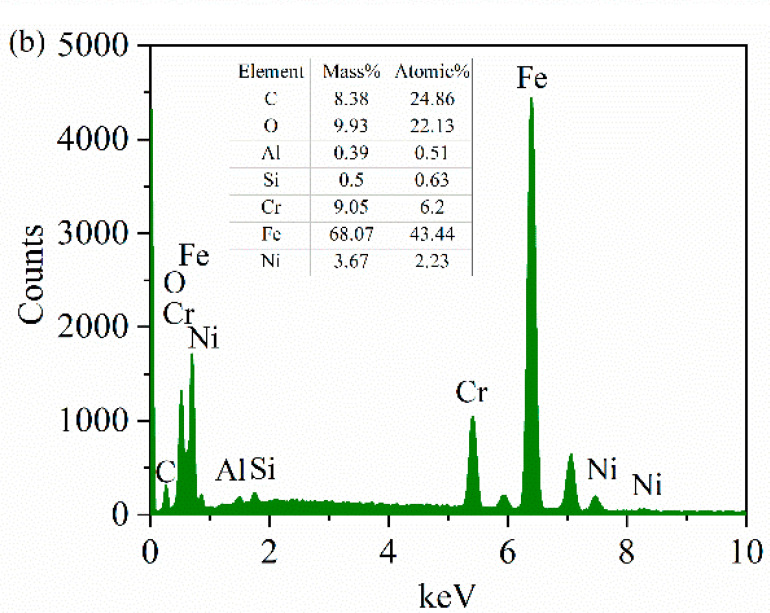

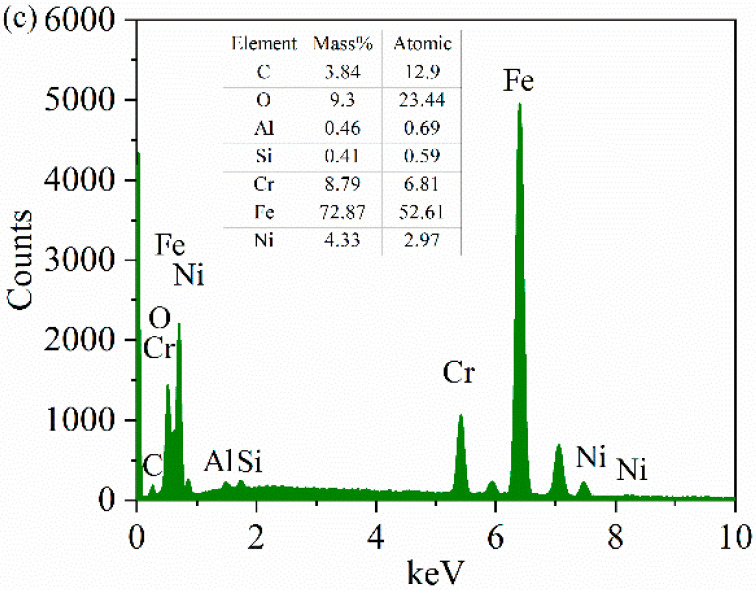
Comparison of EDS spectra of the picosecond textured worn balls: (**a**) rotation radius 15 mm; (**b**) rotation radius 18 mm; and (**c**) rotation radius 22.5 mm.

As mentioned earlier, it is impossible to judge whether there is a material transfer between the disk and the ball in the energy spectrum elements on the surface of the plate sample. From the energy spectra of spheres in Figure 11b,c and Figure 12b,c, we can see that there is an Al element, and the mass fraction of the Ni element significantly increases. It can be boldly inferred that the material of the nanosecond texture plate is transferred to the ball. This further confirms our judgment that the wear debris produced by friction is under the joint action of load, sliding friction speed, collision, vibration and friction heat. A part of the wear debris is captured by the groove, and a part is glued and transferred to the surface of the ball specimen, forming corrugated wear marks to protect the lower specimen and reduce the friction. The Al content in Figure 11b,c is higher than that in Figure 12b,c, indicating that the material transfer on the surface of nanosecond texture is greater under the same rotation radius. This also indirectly shows that the wear rate of nanosecond texture surface is higher than that of picosecond texture under the same rotation radius.

## 4. Conclusions

This paper mainly studies the influence mechanism of different groove collision frequencies on the friction and wear properties of nanosecond groove texture and picosecond groove texture on the surface of 0Cr17Ni7Al stainless steel. Some of the main results are summarized as follows.

1. Nanosecond and picosecond lasers can produce groove textures. However, the surface quality of the groove texture processed by picosecond laser is better. In all experiments, the largest friction coefficient is 0.8425 when the nanosecond texture is at a rotation radius of 22.5 mm. The minimum friction coefficient is 0.7318 at the rotation radius of 15 mm in terms of nanoseconds. However, for the wear rate, the wear rate of nanosecond texture at the rotation radius of 15 mm reached *H* = 13.680 × 10^−4^ mm^3^/N·mm. This wear rate is higher than the previous results of different preparation methods under the same experimental conditions. Not all groove textures have higher wear rates than those without texture at all rotation radii. Therefore, this needs to be fully considered when selecting the groove texture preparation method.
2. The friction coefficients of nanosecond and picosecond groove texture are smaller than that of the untextured surface, which is mainly related to the 30 collisions that occur between ball and groove texture at each cycle of the friction process. On the one hand, during collision between the ball and the groove, while changing the wear debris from sliding friction to rolling friction, groove texture is promoted, making it easier to capture and store the wear particles. On the other hand, the vibration generated in the collision process effectively drives the wear debris to the edge of the wear mark. This promotes the formation of an edge hard-phase peak. The edge hard phase peak plays a role in reducing friction. This is consistent with the previous studies on electrospark and femtosecond preparation methods. It is confirmed that, under the same size parameters and test conditions, the impact mechanism of friction is the same, regardless of the groove texture preparation method.
3. The friction-reducing performance of nanosecond texture and picosecond texture decreases with the increase in rotation radius and decrease in collision frequency. The wear resistance showed a surprising reverse trend. With the increasingly excellent wear resistance, the collision frequency decreases. This is completely different from the previous studies on EDM texture and femtosecond texture. It can be seen from the research that, for the groove texture with different preparation methods, the influence law of collision frequency on friction reduction and wear resistance is different. This discovery is very important and breaks the stereotype of previous research. It has practical guiding significance in engineering applications.
4. From the appearance of oxygen on the surface of the sphere in the energy spectrum, it can be judged that three kinds of wear are concurrent in the friction process: abrasive wear, adhesive wear and oxidation corrosion wear. An Al element is added to the energy spectrum of nanosecond texture and picosecond texture. The mass content of the Ni element is significantly higher than that of the original Ni element on the ball surface, by 0.06%. Therefore, it can be inferred that, during the friction process, the ball collides with the groove texture and transfers the wear debris to the surface of the ball mill mark. Under the joint action of pressure, rotating speed and friction heat, the wear debris forms a wavy friction-reducing area to reduce friction.

Suggestions for future work include:

1. Use CFD finite-element analysis technology, combined with friction and wear test. Confirm the results.
2. A wear prediction model suitable for textured surfaces is proposed to guide practical engineering practices.
3. Add conditions for additional experiments. Parameters such as load, speed and area density are analyzed, to increase the understanding of the influence law of friction and wear.
4. To explore the dynamic measurement of instantaneous friction heat. Accurately evaluate the effect of friction heat on friction and wear.
5. More abundant surface groove texture preparation technologies, such as electrolysis, micromachining and ultrasound, were studied. The advantages and disadvantages of each preparation technology for the surface groove texture were found.
6. Research was conducted on the low-cost, textured green repair of friction surfaces that have been slightly worn.

## Figures and Tables

**Table 1:** Specific parameters of nanosecond laser equipment.

Parameters	Value	Unit
Pulse frequency	50–500	kHz
Laser wavelength	355	nm
Cutting format	700 × 600	mm
Cutting efficiency	800–7000	mm/s
Laser power	15 (max)	W
Comprehensive accuracy	±30	µm

**Table 2:** Specific parameters of picosecond laser equipment.

Parameters	Value	Unit
Pulse frequency	1–2000	kHz
Laser wavelength	1030	nm
Cutting format	700 × 600	mm
Cutting efficiency	800–7000	mm/s
Laser power	20 (max)	W
Comprehensive accuracy	±30	µm

**Table 3:** Parameters of texture samples.

Test Piece	Material	Hardness	Geometric Dimension	Surface Roughness
Upper test ball	9Cr18	64HRC	Φ9.525 mm	0.014 µm
Lower test plate	0Cr17Ni7Al	42HRC	Φ50.8 mm × 6.35 mm	0.05 µm

**Table 4:** Experimental conditions for rotation test.

Specimen Name	Test Radius (mm)	Rotation Speed (r/min)	Load (N)	Time (min)
Nanosecond texture	15	30	10	20
	18	25	10	20
	22.5	20	10	20
Picosecond texture	15	30	10	20
	18	25	10	20
	22.5	20	10	20

**Table 5:** Friction coefficient and wear rate.

Specimen Name	Rotation Radius (mm)	Average Friction Coefficient	Wear Rate $\left(10^{-4}{\mathrm{mm}}^{3}/N\cdot \mathrm{mm}\right)$
Nanosecond texture	15	0.732	13.68
	18	0.755	6.647
	22.5	0.843	4.741
Picosecond texture	15	0.743	7.756
	18	0.793	5.132
	22.5	0.816	3.342
Untexture	15	0.895	5.219
	18	0.870	6.352
	22.5	0.867	5.140

**Table 6:** Collision parameters with different rotation radius.

Rotation Radius (mm)	Time (min)	Speed (r/min)	Number of Grooves per Turn	Total Number of Friction Turns	Total Times of Friction and Collision
15	20	60	30	1200	36,000
18	20	50	30	1000	30,000
22.5	20	40	30	800	24,000

**Table 7:** Chemical composition and mass fraction of friction pair.

Specimen Name	Material	C	Si	Mn	P	S	Ni	Cr	Al
Plate	0Cr17Ni7Al	0.09	1.0	1.0	0.04	0.03	6.5~7.75	16~18	0.75~1.5
Ball	9Cr18	0.9~1.0	0.8	0.8	0.04	0.03	0.06	17~19	-
